# PV generation and load profile data of net zero energy homes in South Australia

**DOI:** 10.1016/j.dib.2019.104235

**Published:** 2019-07-08

**Authors:** Vanika Sharma, Mohammed H. Haque, Syed Mahfuzul Aziz

**Affiliations:** School of Engineering, University of South Australia, Mawson Lakes, SA 5095, Australia

**Keywords:** Net zero energy home, PV generation, Renewable energy system, Residential load demand, Rooftop PV system

## Abstract

This paper presents the hourly Photovoltaic (PV) generation and residential load profiles of a typical South Australian Net Zero Energy (NZE) home. These data are used in the research article entitled “Energy Cost Minimization for Net Zero Energy Homes through Optimal Sizing of Battery Storage System” Sharma et al., 2019. The PV generation data is derived using the publicly accessible renewable ninja web platform by feeding information such as the region of interest, PV system capacity, losses and tilt angle. The raw load profile data is sourced from the Australian Energy Market Operator (AEMO) website, which is further processed and filtered to match the household load requirement. The processing of data has been carried out using Microsoft Excel and MATLAB software. The experimental method used to obtain the required data from the downloaded raw dataset is described in this paper. While the data is generated for the state of South Australia (SA), the method described here can be used to produce datasets for any other Australian state.

**Table:** Specifications table

Subject area	*Electrical Engineering*
More specific subject area	*Renewable energy systems, photovoltaic system, battery energy storage system, Net Zero Energy home*
Type of data	*Excel file*
How data was acquired	*Publicly available load and PV generation data processed using a MATLAB program*
Data format	*Raw, Processed, Filtered*
Experimental factors	*Realignment of original data from UTC to South Australian time*
Experimental features	*Scaling down of the load profile data for a region in the state of South Australia to that of a single residential dwelling; filtering hourly PV generation data for a single household*
Data source location	*The Australian state of South Australia*
Data accessibility	*Data is provided with this article*
Related research article	*V. Sharma, M. H. Haque, S. M. Aziz, Energy Cost Minimization for Net Zero Energy Homes through Optimal Sizing of Battery Storage System, Renewable Energy, Volume 141, 2019**[1]**.*

**Table:** 

**Value of the data** • This dataset serves as a repository of Photovoltaic (PV) generation and electricity usage pattern of a residential dwelling in South Australia. • The reported hourly generation and load profile for a full year can be used to devise energy management strategies for homes with PV and battery storage systems. • The dataset are useful to determine the optimal battery size for various system configurations with a view to reduce the net cost of energy to the home owner. • Researchers can use the dataset to replicate the results reported in the original article and compare the efficacy of the presented model [1] with other methods. • The dataset can be used to determine the economic value of small-scale PV systems as well as to study the impacts of high residential PV penetration on the voltage profile of distribution lines.

## 1 Data

**Fig. 1:**
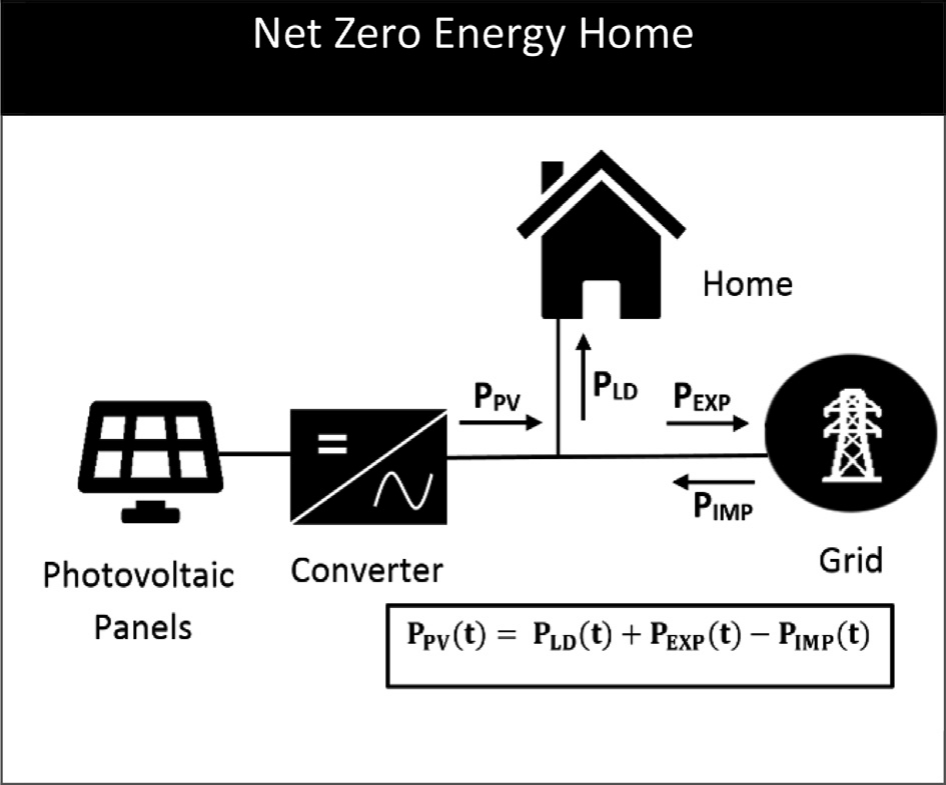
Configuration of a grid connected NZE home with rooftop PV system.

Fig. 1 shows the general configuration of a Net Zero Energy (NZE) home. The data is supplied in two separate Excel files: one containing raw half-hourly load data for a region in South Australia, and the other containing PV generation and load data scaled down for a single home. Examining the second file will reveal that it consists of three variables: (1) PV power generation of a 3 kW_p_ system (P_PV_), (2) residential load demand (P_LD_) of a typical home, and (3) ambient temperature. Both the PV generation and load data are represented in kW. The data are specifically filtered for the state of South Australia (SA) and represent hourly data for a full year. The data contain information related to PV generation pattern of SA region, for example, the minimum PV generation occurs during the middle of the calendar year due to winter. The load data provide insight into the electricity usage pattern of South Australian homes. For example, electricity demand peaks at certain times in the early and late days of a year due to high summer temperatures, which increases the air-conditioning load.

**Fig. 2:**
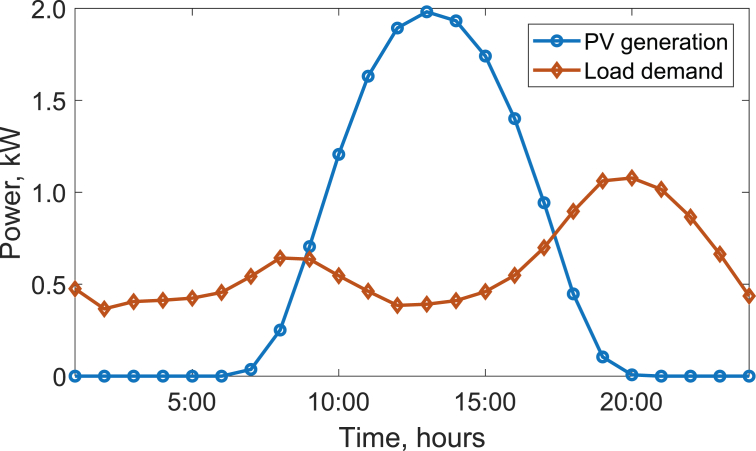
Average daily PV generation and load profile of a typical SA household for 2015.

Fig. 2 illustrates the average daily PV generation and load profile of a typical SA household for the year 2015. This clearly demonstrates significant mismatch between the PV generation and load patterns, which provides the justification for using battery storage with PV systems [1]. In Fig. 1, the exported power (P_EXP_) and the imported power (P_IMP_) represent the amount of power exchanged between the home and the grid due to the mismatch. The power balance equation at the point of common coupling is shown in Fig. 1. When the PV generation is higher than the load demand then *P*_*EXP*_*(t) > 0* and *P*_*IMP*_*(t) = 0*. When the PV generation is lower than the load demand then *P*_*EXP*_*(t) = 0* and *P*_*IMP*_*(t) > 0*. Because the dataset relate to a NZE Home, the overall PV generated energy and the energy consumed by the home is the same over a year. Therefore, the annual exported energy is the same as the annual imported energy. The method used to produce the data is given in the next section.

## 2 Experimental design, materials, and methods

The method to produce PV generation and residential load profile is given below.

### 2.1 PV generation data

Hourly PV generation and ambient temperature data have been derived using web platform Renewable ninja [2]. To generate the data the following information is supplied to the system:

• Location of the PV system
• Target PV system capacity
• PV system loss (including converter losses)
• Tilt angle
• Azimuth angle

These factors can be varied according to system requirement. Additional features such as tracking type can be included. The data presented in this article is an hourly PV generation profile of the year 2015. Note that the data downloaded from this source is for the UTC time zone. However, South Australian (SA) time is 10.5 hours ahead of UTC time during the month of January. Therefore, a few rows of data from the bottom of the generated file need to be shifted to the top rows to align with South Australian time.

### 2.2 Residential load profile

An hourly residential load profile for a typical South Australian home has been obtained by scaling down the publicly available load data provided by Australian Energy Market Operator [3]. This data has been filtered and scaled down using the steps given below:

1. From the AEMO website [3], select Data→Metering→Load profiles.
2. Select the calendar year (2015) to download the load profile for that year.
3. There are two types of load profiles: Net System Load Profile (NSLP) and Controlled Load (*cload*). Choose NSLP.
4. Among the various profile areas in NSLP, select UMPLP area. In this area, electricity is supplied by the South Australian retailer *SA Power Networks* [4]. This would provide the regional load profile data of SA. This data is supplied in one of the accompanying Excel files.
5. The downloaded Excel file contains half-hourly load demand of the selected year. Convert the half-hourly data to hourly load demand using the ‘Average’ function in Excel or using the MATLAB code attached with this article.
6. Now the hourly load demand for the selected area of SA is obtained. This load pattern closely resembles that of a residential dwelling in SA [5].
7. To obtain the load profile of a single residential dwelling in SA, scale down each of the hourly load data points using the following formula:

$$x_{i}=\;aX_{i}$$

where, $x_{i}$ is the scaled down load data at time ‘i’, $X_{i}$ is the downloaded load data which is being scaled, a is the scaling factor and equates to the ratio of annual energy consumption of the NZE home (5,210.9 kWh) to the total annual energy consumption in the downloaded data. The annual consumption of a NZE home is taken as 5,210.9 kWh, which is only 1.28% higher than a typical SA home's energy consumption of 5,145 kWh [6]. This annual consumption is selected because it is a NZE home with a 3 kW_p_ rooftop PV system which can produce 5,210.9 kWh energy annually. The MATLAB code attached with this article can be used to scale the data.
8. The result of Step 7 is the hourly load profile data of a typical SA home for the selected year (2015). This data is supplied in the second Excel file accompanying this paper. For convenience, the dates and times have been included. This load profile has been used for the modelling and analysis reported in [1].

The above steps can be used to generate household load and PV generation data for other Australian states.
